# Pericardial and mediastinal fat-associated lymphoid clusters are rapidly activated in an alkane-induced model of systemic lupus erythematosus

**DOI:** 10.1093/discim/kyad017

**Published:** 2023-09-25

**Authors:** Karolina Bentkowska, Alex Hardgrave, Nadia Iqbal, Laura Gresty, Bethany Marsden, Sheila Macharia, Lucy Jackson-Jones

**Affiliations:** Division of Biomedical and Life Science, Lancaster University, Lancaster, UK; Division of Biomedical and Life Science, Lancaster University, Lancaster, UK; Division of Biomedical and Life Science, Lancaster University, Lancaster, UK; Division of Biomedical and Life Science, Lancaster University, Lancaster, UK; Division of Biomedical and Life Science, Lancaster University, Lancaster, UK; Division of Biomedical and Life Science, Lancaster University, Lancaster, UK; Division of Biomedical and Life Science, Lancaster University, Lancaster, UK

**Keywords:** lupus, pleuritis, SLE, pristane, fat-associated lymphoid clusters

## Abstract

Systemic lupus erythematosus (SLE) is an autoimmune disease predominated by auto-antibodies that recognise cellular components. Pleural involvement is the most common SLE-related lung disease. Natural antibodies are rapidly secreted by innate-like B cells following perturbation of homeostasis and are important in the early stages of immune activation. The serous cavities are home to large numbers of innate-like B cells present both within serous fluid and resident within fat-associated lymphoid clusters (FALCs). FALCs are important hubs for B-cell activation and local antibody secretion within the body cavities. Patients with SLE can develop anti-phospholipid antibodies and in rare situations develop alveolar haemorrhage. Utilising delivery of the hydrocarbon oil pristane in C57BL/6 mice as a model of SLE we identify a rapid expansion of pleural cavity B cells as early as day 3 after intra-peritoneal pristane delivery. Following pristane delivery, pericardial B1 B cells are proliferative, express the plasma-cell surface marker CD138, and secrete both innate and class-switched antibodies highlighting that this cavity niche may play an unrecognised role in the initiation of lupus pleuritis.

## Graphical Abstract

**Graphical Abstract:**
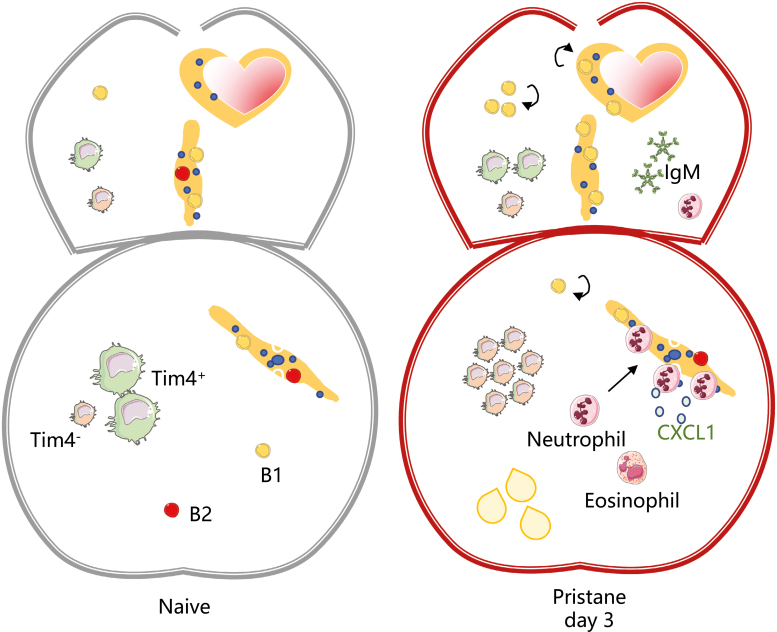

## Introduction

Systemic lupus erythematosus (SLE) is an auto-immune disease characterised by the production of systemic anti-nuclear antibodies, development of arthritis, immune complex-mediated glomerulonephritis as well as pericardial and pleural inflammation. The mechanism of pleural involvement in SLE is under-investigated; however, 45–60% of SLE patients experience pleuritic pain and pleural involvement is one of the most common SLE-lung-related diseases [1]. Overproduction of natural antibodies against self-antigens is a characteristic feature of autoimmune diseases such as SLE. Furthermore, 30–40% of SLE patients are positive for the presence of anti-phospholipid antibodies [2]. Previous studies have also confirmed the presence of anti-nuclear antibodies within pleural fluid of patients with SLE and found these to be at a higher titre within pleural fluid than the serum suggesting a local accumulation or production [3]. In mice, the serous cavities, including the pleural cavity are predominated by B1 B cells [4] and are key sites at which natural antibodies are detected following their local secretion from fat-associated lymphoid clusters (FALCs) [5, 6]. Omental FALCs (previously known as milky spots) are also important sites providing protection to the serous cavities in the context of inflammation [7, 8], infection [9, 10], and cancer [11]. In humans, the frequency of B cells within peritoneal lavage is lower than that of mice [12], which may reflect the larger capacity of the human omentum to house B cells given the size of this apron of adipose. The alkane tetramethylpentadecane (TMPD, commonly known as pristane) is a hydro-carbon oil which has been shown to model SLE following injection *in vivo* in animal models [13].

Studies utilising pristane mainly focus on the detection of natural antibodies in the serum typically weeks after pristane delivery when the response becomes systemic [14–18]. In different strains of mice, pristane induces distinct responses, for example, BALB/c mice go on to develop arthritis whereas C57BL/6 mice instead develop pulmonary complications including diffuse alveolar haemorrhage [13]. The baseline number and activation of FALCs within the pericardium and mediastinum of mice [5] also differs by strain, with those on the C57BL/6 background being more strikingly activated during allergic inflammation than BALB/c. A role for Immunoglobulin M(IgM) in the development of thoracic complications of SLE has been shown previously; B-cell deficient μMT^−/−^ mice that are resistant to development of diffuse alveolar haemorrhage become susceptible following infusion of IgM [19]. As FALCs within the pericardium and mediastinum are key sites for local IgM secretion within the pleural cavity [5] and expansion of mediastinal FALCs has been shown in an MRL/MpJ-lpr autoimmune mouse model of SLE [20] we aimed to determine whether these structures are activated during pristane induced pleuritis.

Studies of pristane exposure commonly assess systemic auto-antibody responses in the weeks to months following exposure and as such, the early immune response within the pleural cavity remain relatively elusive. To better understand the pleural cavity response to pristane we compared the early events occurring at this site with those in the peritoneal cavity which is the site of pristane delivery. We found higher numbers of B cells within the pleural cavity by day 3 following i.p. delivery of pristane with increased proliferation of B1 B cells within both pleural fluid and FALCs. In contrast to the omentum, which did not show increased antibody secretion, there was a significant increase in total IgM, IgG2a, and IgG1 antibody released by the pericardial and mediastinal FALCs at day 3 following exposure to pristane. Furthermore, we detected an increase in natural IgM antibodies that recognise phospho-lipids within lavage fluid from the pleural cavity following pristane delivery.

## Methods and materials

### Animals

Experiments performed at Lancaster University were conducted in accordance with the Animals (Scientific Procedures) Act, UK 1986 under license granted by the Home Office (UK) following prior approval by local animal welfare and ethical review body. Experiments were performed using female C57BL/6J mice aged 8–12 weeks, animals were purchased from Charles River or were the wildtype offspring of genetically modified animals that were bred and maintained under specific pathogen-free conditions at Lancaster University.

### *In vivo* procedures

Mice were injected i.p. with 300 μl of the hydrocarbon oil pristane (Sigma) or left naïve. Pleural and peritoneal exudate cells (PLEC and PEC) were isolated by flushing murine cavities with a minimum of 2 ml of RPMI 1640 (Sigma). Cell pellets were isolated by centrifugation and the first 2 × 1 ml of lavage fluid was stored at −20 to −80°C for further analysis. Omentum, pericardial, and mediastinal adipose tissues were isolated following lavage, weighed, and in select experiments omentum and mediastinal adipose were cultured *ex vivo* in 500 μl of complete RPMI 1640 (sigma) containing 10% Foetal calf serum (Sigma), 1% (v/v) Penicillin Streptomycin (Gibco) for 2 h at 37°C, 5% CO_2_ and the culture supernatant stored as above.

### Flow cytometry

Murine cells were stained to distinguish live versus dead using Zombie aqua (Biolegend), blocked with anti-murine CD16/32 (clone 2.4G2, Biolegend) and 10% rat and mouse serum (Sigma) and stained for cell surface markers (see Supplementary Table 1 for list of antibodies used), following staining cells were washed using FACs buffer (2% bovine serum albumin [BSA], 2 mM Ethylenediaminetetraacetic acid [EDTA], Phosphate Buffered Saline [PBS]). In select experiment cells were fixed and permeabilised using eBioscience-FoxP3 fixation and permeabilization buffer and incubated with anti-Ki67 antibodies for a minimum of 30 min prior to washing with acquisition. All samples were acquired using a Beckman Coulter Cytoflex and analysed with FlowJo software (Tree Star).

### Wholemount immunofluorescence staining

Murine omentum, pericardial, and mediastinal adipose tissues were fixed for 1 h on ice in 10% Neutral Buffred Formalin (NBF) (Fisher or Sigma) and then permeabilized in PBS 1% Triton-X 100 (Sigma) for 20 min at room temperature prior to staining with primary antibodies for 1 h in PBS 0.5% BSA 0.5% Triton (Sigma). Antibodies used are listed in Supplementary Table 1.

### Microscopy

After mounting with Fluoromount G (Invitrogen), confocal images were acquired using a Leica Stellaris 5 laser scanning confocal microscope or a Zeiss LSM880, analysis was performed using Image, J. To calculate the area of FALCs stained with antibodies of interest, the perimeter of the FALC was delimited manually and a fixed threshold for IgM, F4/80, and Ki67 fluorescence was set.

### Detection of cytokines, chemokines, and antibodies

A mix and match Murine anti-viral Legendplex array (Biolegend) was used to detect CXCL1 and a 6-plex murine isotyping panel was used to detect total IgM, IgG2a, IgG1, and IgA within tissue culture supernatants following the manufacturer’s instructions with the modification that half of the suggested beads and reagents were used per assay; following prior optimisation. For the detection of IgM-recognising phospholipid species, 5 μg/ml of oxidised low-density lipoprotein (oxLDL) (Invitrogen), 1-palmitoyl-2-(5ʹ-oxo-valeroyl)-*sn*-glycero-3-phosphorylcholine (POVPC) (Avanti lipids), oxidised-1-palmitoyl-2-arachidonoyl-*sn*-glycero-3-phosphorylcholine (OxPAPC) (Avanti lipids) were coated overnight on 96-well high-binding ELISA plates (Costar), washed with Tris-buffered Saline + 0.05%Tween, blocked using 2% (w/v) BSA; Sigma and incubated with pleural lavage fluid overnight at 4°C prior to detection of bound IgM with a goat anti-mouse IgM-horse radish peroxidase (HRP) (1020-05 Southern Biotech) for 1 h at 37°C. 50 μl of 3,3ʹ,5,5ʹ-Tetramethylbenzidine (Biolegend) was added, allowed to develop, and stopped with 50 μl 0.18M H_2_SO_4_. Plates were read at 450 nm on Tecan Infinite 200PRO plate reader.

### Statistical analysis

Power calculations showed that for our most commonly measured parameters (cell number) six mice per group provide sufficient power (90%) to detect at least a 1.5-fold difference between the groups, which we regard as an acceptable cut-off for identifying important biological effects. No randomisation and no blinding was used for the animal experiments. Whenever possible, the investigator was partially blinded for assessing the outcome (confocal analysis). All data were analysed using Prism 9 (Graphpad Prism, La Jolla, CA, USA). Statistical tests performed, sample size, and number of repetitions for each data set, are described within the relevant figure legend.

## Results and discussion

**Figure 1:**
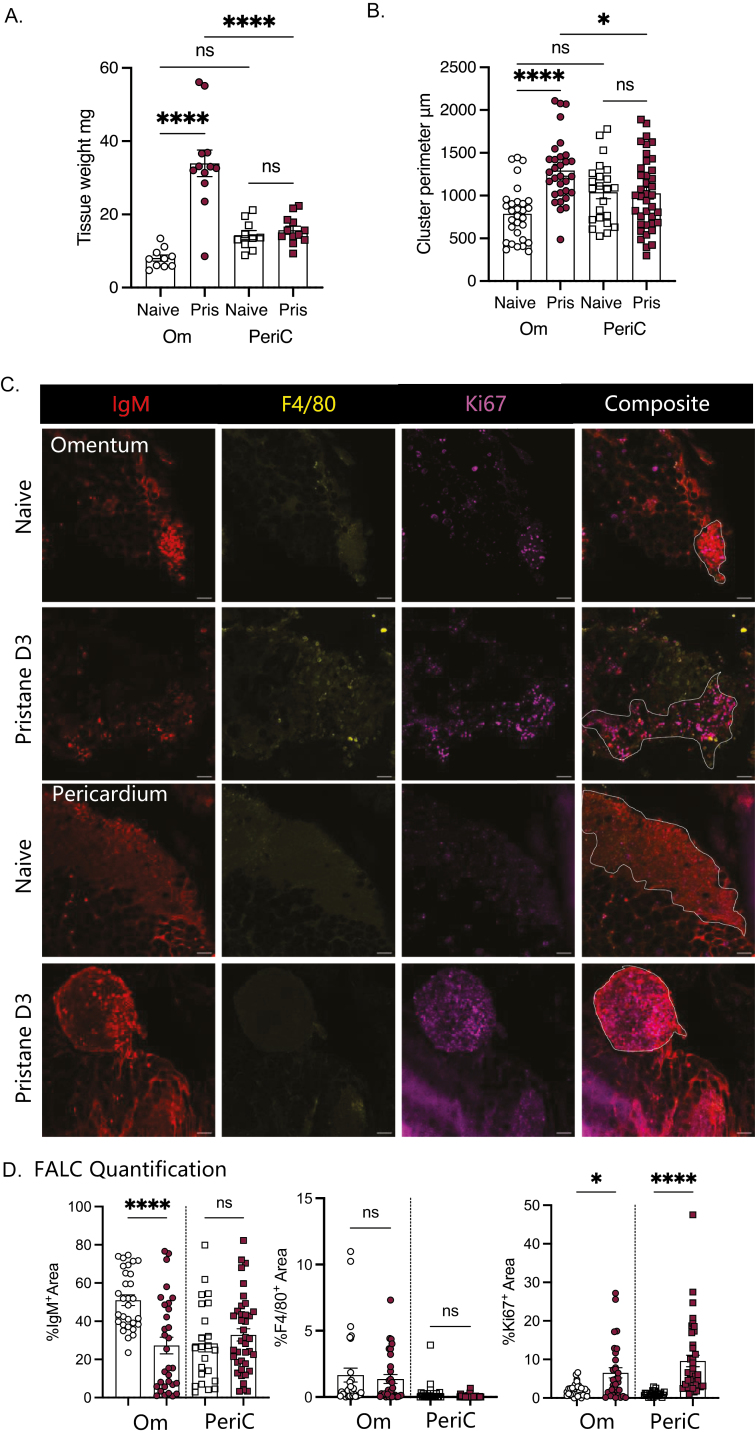
FALCs are activated in response to intra-peritoneal pristane delivery. C57BL/6 mice were injected i.p. with 300 μl pristane or left naive, 3 days later omentum and pericardium were isolated and weighed (A), whole-mount immuno-fluorescence confocal microscopy was undertaken on omenta and pericardia to determine cluster perimeter (B), IgM (Red), F4/80 (Yellow) and Ki67 (magenta) expression (C), area of expression per cluster was quantified using Image J (D). Data in A pooled from three independent experiments, *n* = 3–5 mice per group. Data in B and D pooled from two independent experiments, data points represent individual FALCs from 3 to 5 mice per group. Data in C representative of two experiments, *n* = 3–5 mice per group. Student’s T-Test, *P < 0.05, ****P ≤ 0.0001, ns = non-significant, scale bar = 50 μM.

FALCs are key sites of immune orchestration within the serous cavities, in order to determine the early immune response to pristane within the cavities we first quantified key immune cell populations within FALC containing adipose depots (omentum, pericardium, and mediastinum) of both the peritoneal (site of delivery) and pleural (distal site) cavities. As we were interested to better understand the initiation of inflammation within the pleural cavity, we utilised C57BL/6 mice that have been shown to develop pulmonary complications following delivery of pristane. To determine the role of FALCs in co-ordinating the immune response within the body cavities following pristane exposure we injected a low volume (300 μl) of pristane into the peritoneal cavity (to limit potential for direct leakage of pristane from the peritoneal into the pleural cavity) and isolated the omental and pericardial adipose tissues after 3 days. Weighing of the tissues revealed an increase in omental but not pericardial adipose weight (Fig. 1A), this increase in weight appeared to be mediated by an expansion in the size of FALCs within the tissue as determined via analysis of cluster perimeter using Image J following whole-mount immuno-fluorescence analysis of fixed tissues (Fig. 1B). To determine which immune cells may be responsible for the early differential expansion of FALCs within the omentum and pericardium following pristane exposure we analysed the % area of expression with individual FALCs via whole-mount immuno-fluorescence staining for IgM (B-lymphocytes, red), F4/80 (serous cavity macrophages, yellow), and Ki67 (proliferation, magenta) (Fig. 1C). There was a significant reduction in the % area of IgM within clusters of the omentum at day 3 following pristane delivery, suggesting either a reduction in the number of B cells present within the tissue or an indication that the B cells within this tissue had undergone class switch to express a different antibody isotype (Fig. 1D). In contrast, there was no significant difference in the percentage area of IgM expression within the pericardial FALCs after pristane delivery (Fig. 1C and D). This data suggested that B cells were not the main cell type responsible for early FALC expansion within the omentum. Peritoneal macrophages have been shown to migrate to the omentum during infection of the peritoneum [6, 9, 10] as such, we also assessed F4/80 within FALCs of the omentum and pericardium and found no significant increase in the percentage area of expression, of this serous cavity macrophage receptor at 3 days after pristane injection (Fig. 1D). Percentage area of expression of F4/80 was variable mouse to mouse and there was a trend in one of three experiments performed towards an increase in macrophage recruitment within the omentum but this trend was not seen in the pericardium. In contrast to these cell-type defining markers, we saw a significant increase in percentage area of expression of both omental and pericardial FALCs for the nuclear cell cycle protein Ki67 which is an accepted readout of cellular proliferation (Fig. 1C and D).

**Figure 2:**
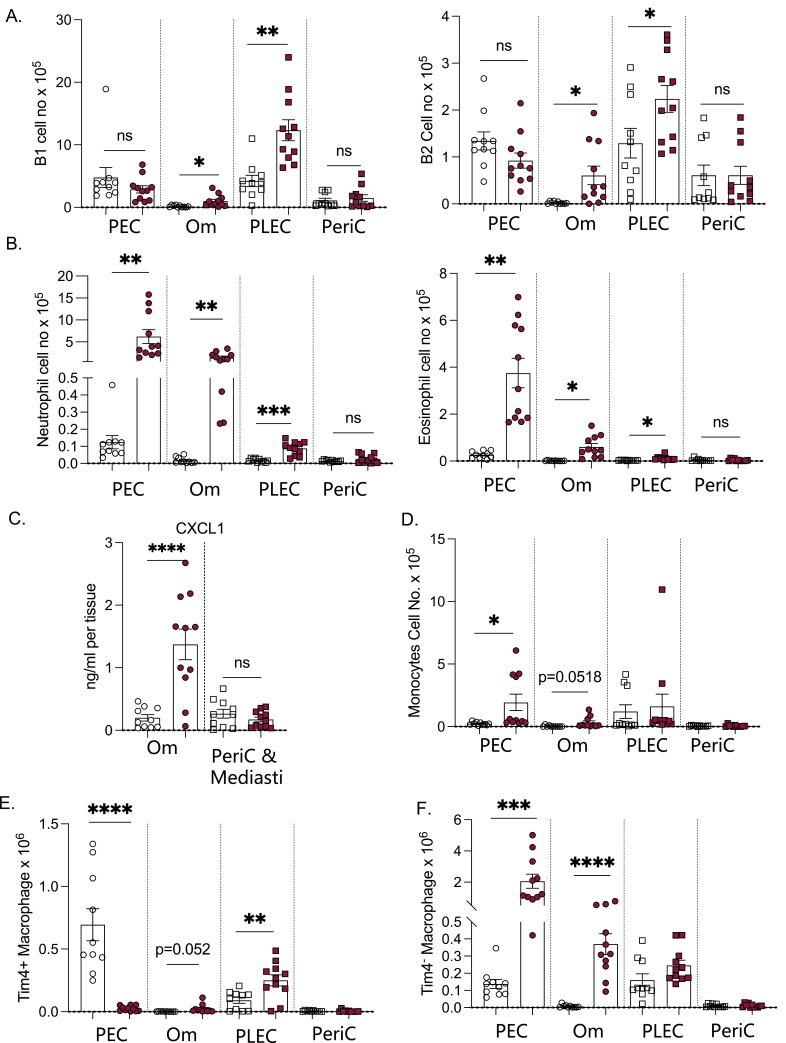
Pristane delivery induces differential immune cell changes within the peritoneal and pleural cavities. C57BL/6 mice were injected i.p. with 300 μl pristane (maroon) or left naïve (white), 3 days later Peritoneal lavage, Pleural lavage, omentum, pericardium, and mediastinum were isolated and tissues weighed. Omenta and pericardium were digested and assessed by flow cytometry alongside PEC and PLEC (A–B, D–E) for the presence of immune cells (Live, singlets, CD45^+^) including B1 B cells (Ly6G^−^CD19^+^MHCII^+^CD11b^+^), B2 B cells (Ly6G^−^CD19^+^MHCII^+^CD11b^−^), neutrophils (Ly6G^−^), eosinophils (Ly6G^−^SigF^+^SSCA^hi^), monocytes (Ly6G^−^CD19^−^TCRβ^−^SigF^−^Ly6C^+^), Tim4 + macrophages (Ly6G^−^CD19^−^TCRβ^−^SigF^-^Ly6C^−^F4/80^+^CD11b + Tim4^+^), and Tim4^−^ macrophages (Ly6G^−^CD19^−^TCRβ^−^SigF^−^Ly6C^−^F4/80^+^CD11b + Tim4^−^). Omenta and mediastina were placed in culture at 37°C 5%CO_2_ for 2 h and total amount of CXCL1 secreted into the culture supernatant within 2 h was quantified using a legendplex array, total CXCL1 secreted from whole FALC containing tissue was then calculated using whole tissue weights within each cavity (C). Data pooled from three independent experiments *n* = 3–5 mice per group per experiment, Student’s T-Test, *P < 0.05, **P < 0.01, ***P ≤ 0.001, ****P ≤ 0.0001., Student’s T-Test, *P < 0.05, **P < 0.01, ***P ≤ 0.001, ****P ≤ 0.0001.

To further confirm which cells are responsible for expanded cluster size within the omentum following pristane delivery we assessed using multi-parameter flow cytometry the presence of CD45^+^CD19^+^MHCII^+^CD11b^+^ B1 B cells, CD45^+^CD19^+^MHCII^+^CD11b^−^ B2 B cells, CD45^+^CD19^−^Ly6G^-^SigF^+^SSC-A^hi^ Eosinophils, CD45^+^CD11b^+^Ly6G^+^ neutrophils, CD45^+^CD19^-^Ly6G^-^SigF^-^TCRβ^-^Ly6C^+^ monocytes and CD45^+^CD19^−^Ly6G^−^TCRβ^-^SigF^-^Ly6C^−^CD11b^+^F4/80^+^Tim4^+/−^macrophages within PEC, digested omentum, PLEC and digested pericardium of naïve mice and those exposed 3 days earlier to 300 μl of intra-peritoneal pristane. There was a trend towards reduced numbers of both B1 and B2 B cells within the PEC with a significant increase found within the omentum (Fig. 2A), in contrast to the peritoneal cavity, there was a significant increase in the numbers of B1 and B2 B cells within pleural lavage at Day 3 following pristane injection but no corresponding increase in the pericardium. This data suggests that B cells contribute to differential expansion of FALCs following pristane delivery. The increase in B cells within the omentum was not sufficient to account for the increased cluster size noted during perimeter analysis (Fig. 1B) however we noted a striking increase in the number of eosinophils and neutrophils within the omentum but not the pericardium (Fig. 2B). We have previously shown that the omentum is a key site for neutrophil recruitment during peritonitis [8], to investigate whether the mechanism via which neutrophils are recruited to omental FALCs after pristane exposure may be similar to that during peritonitis we assessed the release of the neutrophil recruitment chemokine CXCL1 and found significantly increased CXCL1 release from the omentum during 2 h of culture when isolated from pristane exposed compared to naïve mice (Fig. 2C). In contrast, there was no increased CXCL1 release from the pericardium and no significant recruitment of neutrophils to this site suggesting that CXCL1 release may be a factor in the recruitment of neutrophils to the omentum in this model. There was a small increase in the number of monocytes within the PEC at Day 3 following pristane delivery and a trend towards an increase in the omentum but no measurable difference in number within the PLEC or pericardium (Fig. 2D). The macrophage disappearance reaction is a phenomenon that characterises the stark absence of large cavity macrophages within the serous cavities rapidly following induction of inflammation [21]. Using a combination of CD11b, F4/80, and Tim4 we were able to define two major populations of macrophages that could be detected in all four sites of interest. As reported previously, the majority of F4/80 high large cavity macrophages (LCM) within naive serous cavities were found to be Tim4^+^, this population was absent in the peritoneal cavity at day 3 after pristane delivery and replaced by an expanded Tim4^−^ population (Fig. 2E and F). A small increase in Tim4^+^ macrophages was found within the omentum but this did not reach significance whereas a large increase in Tim4^−^ macrophages was seen at this site (Fig. 2F) mirroring previous studies that found Tim4^−^ cells at this site to be of bone marrow origin [11], as well as omental accrual of equivalent CD102^−^ inflammation-elicited macrophages [22]. A small but significant increase in the number of Tim4^+^ LCM within the pleural cavity was found at 3 days after pristane delivery (Fig. 2D). It does not appear that the increase in the number of Tim4^+^ macrophages found within the pleural cavity is due to their local proliferation as analysis of the cell cycle protein Ki67 within Live, CD45^+^CD19^−^CD11b^+^ cells that are MHCII^low^ shows no significant increase in expression at either the population or per cell level (data not shown). It would be interesting to investigate in future experiments whether any of the peritoneal macrophages that are lost following pristane injection migrate via the diaphragm to account for this increase found in the pleural fluid. Loss of some of the Tim4^+^ cells within the peritoneal space following pristane injection can be accounted for via migration to the omentum however this by no means accounts for the total loss, indeed as shown in *E. coli* infection experiments [10] we also saw clots of cells or granulomas within the cavity at this time point, however, we have not yet investigated the cellular composition of such.

**Figure 3:**
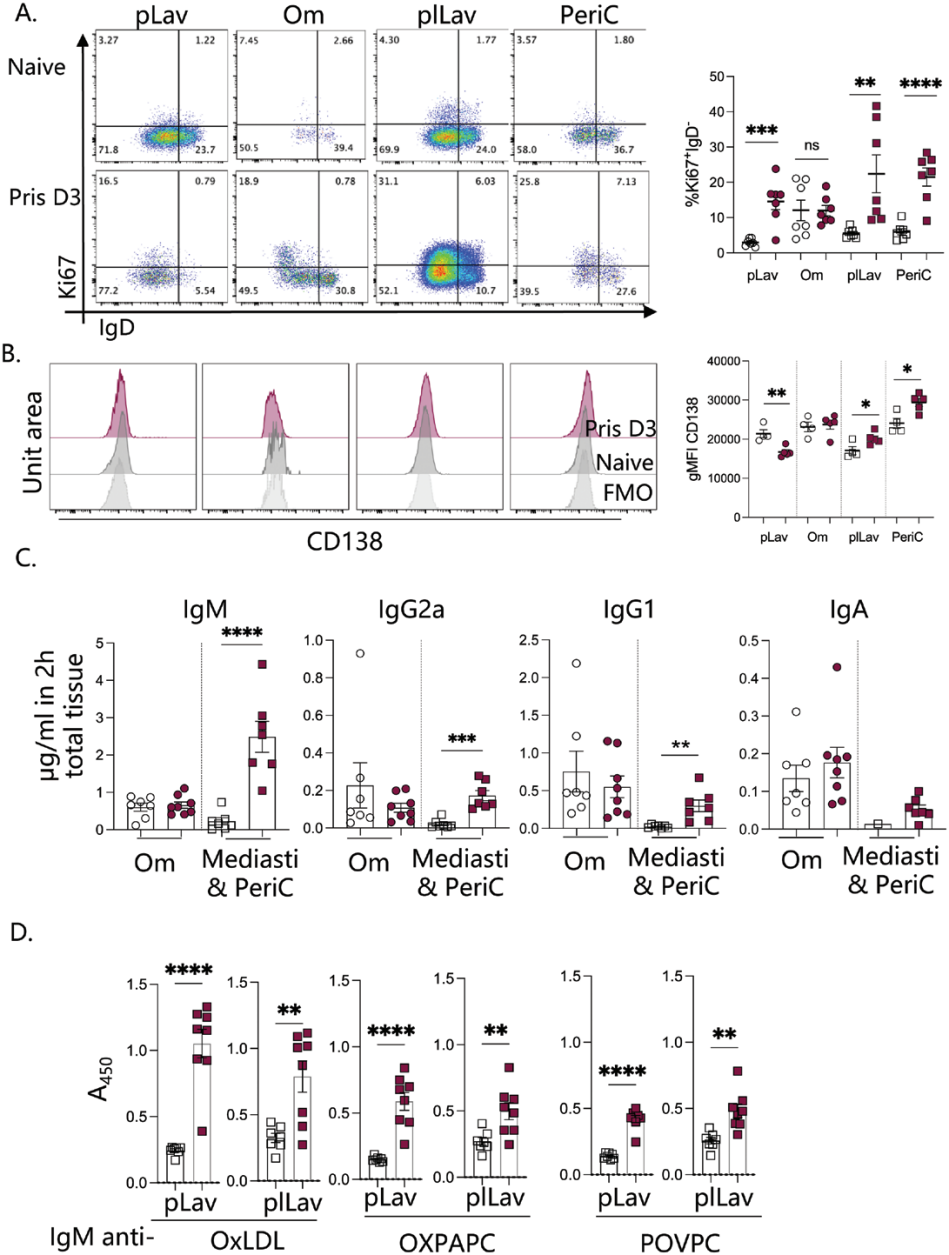
Pleural FALCs produce antibodies in response to intra-peritoneal pristane delivery. C57BL/6 mice were injected i.p. with 300 μl pristane or left naive, 3 days later Peritoneal lavage, Pleural lavage, omentum, pericardium, and mediastinum were isolated and tissues weighed. Omenta were dissected into two and each half was weighed; half of the omentum and pericardium were digested and assessed by flow cytometry alongside PEC and PLEC (A and B) for the expression of Ki67, IgD, and CD138 within B1 B cells (Live, singlets, CD45 + CD19^+^MHCII^+^CD11b^+^). Omenta and mediastina were placed in culture at 37°C 5%CO_2_ for 2 h and total amount of IgM, IgG2a, IgG1, and IgA secreted into the culture supernatant within 2 h were quantified using a Legendplex array, total antibody amount secreted from whole FALC containing tissue was then calculated using whole tissue weights within each cavity (C). Cell-free peritoneal and pleural lavage was assessed for presence of anti-phospholipid IgM antibodies via ELISA (D). Data pooled from two (A, C, and D) and three (B) independent experiments *n* = 3–4 per group, only one naïve mediastinum culture sample had detectable IgA. Student’s T-Test, *P < 0.05, **P < 0.01, ***P ≤ 0.001, ****P ≤ 0.0001.

We next assessed the phenotype of B1 B cells within the peritoneal and pleural cavities and their integral FALC-containing adipose tissues. B1 cells from the PEC, digested omentum, PLEC, and digested pericardium of naïve mice and those exposed 3 days earlier to 300 μl of intra-peritoneal pristane were assessed via flow cytometry to determine nuclear levels of Ki67, the immature B cell surface marker IgD (Fig. 3A) and the plasma-cell surface marker CD138 (syndecan-1) (Fig. 3B). There was a significant increase in the expression of Ki67 by IgD^-^ B1 cells within the PEC, PLEC, and pericardium at 3 days after pristane exposure, indicating that these activated cells were actively progressing through cell cycle. B1 cell proliferation may account for the increased Ki67 detected within the pericardium following pristane exposure (Fig. 1C and D). B1 cells within PLEC and pericardium were also found to have increased per cell expression of CD138 at 3 days following pristane exposure, suggesting that these cells may be actively secreting antibodies (Fig. 3B). This finding was in contrast to B1 cells of the PEC which had reduced expression of CD138 and the omentum in which no difference in CD138 expression was found following pristane exposure (Fig. 3B). We next placed omentum and mediastinum in culture and quantified the secretion of antibodies from the tissues per mg in 2 h to enable calculation of the total amount of antibody released from the total pleural adipose tissues. There was significantly increased secretion of IgM, IgG2a, IgG1, and a trend for increased IgA from pleural FALC-containing tissues at day 3 following pristane exposure (Fig. 3C). IgA was only detected in one of the naïve culture supernatants making statistical analysis unviable. Increased secretion of total IgM, IgG2a, and IgG1 was in contrast to the omentum which did not significantly increase total secretion of these antibody isotypes. Pristane has been shown to cause lymphocyte apoptosis both *in vitro* and *in vivo* [23], as such reduced antibody secretion by the omentum (the FALC containing adipose at the site of injection that was assessed) may in part be due to the loss of viable B cells within this tissue, as supported by reduced IgM staining (Fig. 1D), and no increase in CD138 by flow cytometry. Furthermore, the striking difference in neutrophil numbers within the omentum after pristane delivery in contrast to the failed recruitment to the pericardium and mediastinum (Fig. 2B) may modify the functional capacity of the tissue to secrete antibodies within the 2 h window assessed. Of note, in this work we did not assess *ex vivo* the secretion of antibodies by the mesenteric adipose, an alternate adipose depot within the peritoneum that also houses FALCs [7, 24]; as such, the contribution of mesenteric B cells to the peritoneal antibody environment remains unknown.

Phospholipid antibodies are implicated in the pleural pathogenesis of SLE, as such we next assessed whether we could detect anti-phospholipid antibodies within both the peritoneal and the pleural fluid at day 3 after exposure to pristane. As IgM was the antibody most significantly increased at this timepoint in pleural adipose culture supernatants and IgM having been implicated in the pathology of DAH in C57BL/6 mice we determined via ELISA the presence of IgM recognising the naturally occurring phospholipids oxLDL, oxPAPC, and POVPC within peritoneal and pleural lavage fluid. IgM recognising all three phospholipid species could be detected at day 3 following intra-peritoneal pristane exposure (Fig. 3D) highlighting the local presence of anti-phospholipid antibodies within the pleural cavity early during initiation of lupus pleuritis in a murine model. Given the time frame between stimulation and assessment, it is likely that such increases reflect an expansion of B cells secreting natural antibodies recognising the phosphorylcholine moieties present within all 3 phospholipids analysed and may reflect the presence apoptotic cells within the peritoneal cavity following pristane injection [23].

Collectively, our findings highlight a role of pleural FALCs of the pericardium and mediastinum and the local production of IgM auto-antibodies in the aetiology of lupus-like pleuritis in a C57BL/6 mouse model.

## Supplementary Material

kyad017_suppl_Supplementary_Figure_S1_Table_S1

## Data Availability

The data underlying this article will be shared on reasonable request to the corresponding author.

## Abbreviations

D3: Day 3
FALCs: fat-associated lymphoid clusters
FMO: fluorescence minus one
i.p.: intra-peritoneal
Mediasti: mediastinum
Om: omentum
oxLDL: oxidised low-density lipoprotein
oxPAPC: oxidised-1-palmitoyl-2-arachidonoyl-sn-glycero-3-phosphorylcholine
Peric: pericardium;
PEC: peritoneal exudate cells
pLav: peritoneal lavage
PLEC: pleural exudate cells
plLav: pleural lavage
SLE: systemic lupus erythematosus
TMPD/Pristane/Pris: tetramethylpentadecane
POVPC: 1-palmitoyl-2-(5ʹ-oxo-valeroyl)-*sn*-glycero-3-phosphorylcholine

## Permission to reproduce

The graphical abstract was partly generated using Servier Medical Art, provided by Servier, licensed under a Creative Commons Attribution 3.0 unported license.
