# Supplementary material for: Pericardial and mediastinal fat-associated lymphoid clusters are rapidly activated in an alkane-induced model of systemic lupus erythematosus
Source: Discov Immunol. 2023 Sep 25;2(1):kyad017. doi: 10.1093/discim/kyad017 (PMC10917176; doi:10.1093/discim/kyad017)
Supplement: kyad017_suppl_Supplementary_Figure_S1_Table_S1 [file kyad017_suppl_Supplementary_Figure_S1_Table_S1.pdf]

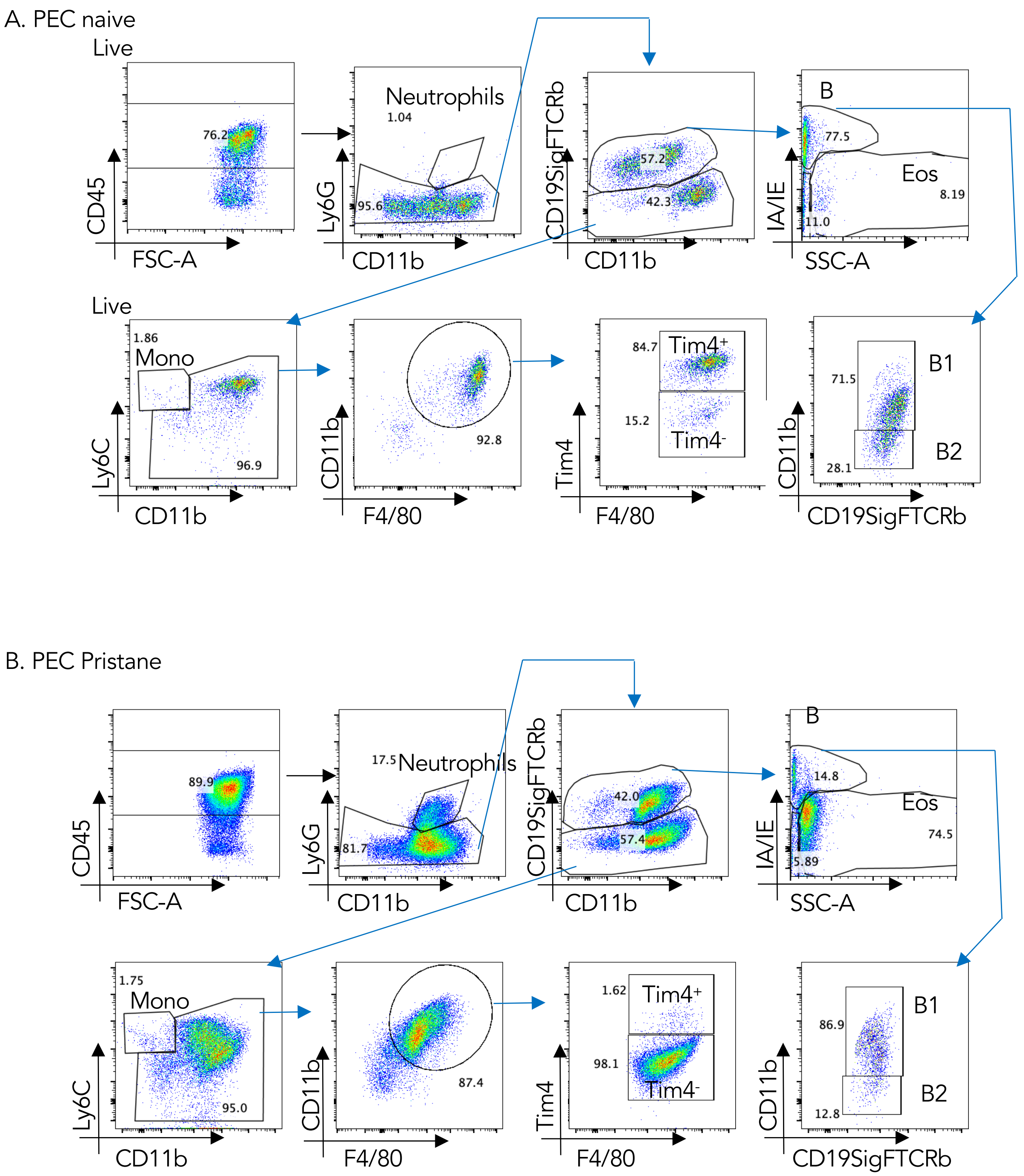


**Supplementary Figure 1. Representative flow cytometry gating strategy**

| Antigen Name | Conjugate | Clone | Manufacturer |
|---|---|---|---|
| IgM | HRP | | Southern Biotech |
| | | | |
| CD11b | PE-Dazzle CF594 | M1/70 | Biolegend |
| CD19 | BV421 | 6D5 | Biolegend |
| Siglec F | BV421 | E50-2440 | BD |
| TCRb | BV421 | H57-597 | Biolegend |
| CD45 | BV650 | 104 | Biolegend |
| F4/80 | PE/Cy7 | BM8 | Biolegend |
| | PE/Cy7 | BM8 | Invitrogen |
| Ki-67 | FITC | REA183 | Miltenyi Biotech |
| Ly6C | Alexa-Fluor 700 | HK1.4 | Biolegend |
| Ly6G | FITC | REA526 | Miltenyi Biotech |
| MHCII IIA/IE | APC/Fire™ 750 | M5/114.15.2 | Biolegend |
| Tim4 | PE | RMT4-54 | Biolegend |
| IgD | PE-Cy7 | 11-26c.2a | Biolegend |
| CD138 | BV605 | 281-2 | Biolegend |
| F4/80 | FITC | BM8 | Biolegend |
| Donkey anti-mouse IgM | Rhodamine Red X | Polyclonal | Jackson Laboratories |
| Ki-67 | VioRed 667 | REA183 | Miltenyi Biotech |

Supplementary table 1. Antibodies used in the study
